# Low-Bandwidth and Compute-Bound RGB-D Planar Semantic SLAM †

**DOI:** 10.3390/s21165400

**Published:** 2021-08-10

**Authors:** Jincheng Zhang, Prashant Ganesh, Kyle Volle, Andrew Willis, Kevin Brink

**Affiliations:** 1 Department of Electrical Engineering, University of North Carolina, Charlotte, NC 28262, USA; arwillis@uncc.edu; 2 Department of Mechanical and Aerospace Engineering, University of Florida, Gainesville, FL 32611, USA; prashant.ganesh@ufl.edu (P.G.); kvolle@ufl.edu (K.V.); 3 Air Force Research Lab, Eglin Air Force Base, Eglin AFB, FL 32542, USA; kevin.brink@us.af.mil

**Keywords:** SLAM, 3D semantic mapping, compression, planar SLAM, shape grammar, semantic segmentation

## Abstract

Visual simultaneous location and mapping (SLAM) using RGB-D cameras has been a necessary capability for intelligent mobile robots. However, when using point-cloud map representations as most RGB-D SLAM systems do, limitations in onboard compute resources, and especially communication bandwidth can significantly limit the quantity of data processed and shared. This article proposes techniques that help address these challenges by mapping point clouds to parametric models in order to reduce computation and bandwidth load on agents. This contribution is coupled with a convolutional neural network (CNN) that extracts semantic information. Semantics provide guidance in object modeling which can reduce the geometric complexity of the environment. Pairing a parametric model with a semantic label allows agents to share the knowledge of the world with much less complexity, opening a door for multi-agent systems to perform complex tasking, and human–robot cooperation. This article takes the first step towards a generalized parametric model by limiting the geometric primitives to a planar surface and providing semantic labels when appropriate. Two novel compression algorithms for depth data and a method to independently fit planes to RGB-D data are provided, so that plane data can be used for real-time odometry estimation and mapping. Additionally, we extend maps with semantic information predicted from sparse geometries (planes) by a CNN. In experiments, the advantages of our approach in terms of computational and bandwidth resources savings are demonstrated and compared with other state-of-the-art SLAM systems.

## 1. Introduction

Many autonomous agent tasks require robust localization and a good representation of the environment, especially when GPS is unavailable. Mobile robots for indoor navigation often operate in structured environments hence indoor navigation focuses more on generating a 2D map. For more complicated tasks, robots need more mobility to operate in more dynamic environments such as slopes, stairs, and tunnels [1,2,3]. In these scenarios, robots are usually equipped with LIDAR and high-quality IMU sensors to estimate the poses and the 3D map [4,5,6]. However, these sensors are quite expensive and relatively fragile. The emergence of modern consumer RGB-D sensors has had a significant impact on the robotic research fields. They are low-cost, low-power, and low-size alternatives to traditional range sensors, such as LiDAR [7]. RGB-D sensors also provide additional depth information which enhances the ability of robots to sense the environment and estimate its structure for navigation. RGB-D cameras have been leveraged for a wide range of research [8,9,10,11].

The motivation of our work is to construct a compact representation of maps that can be efficiently shared among robots. Multi-agent systems have demonstrated various applications, such as self-driving cars, warehouse robots, and so on. These applications require collaborative mapping where each agent needs to share information about their past and current state. The use of SLAM to generate high-quality 2D or 3D maps is a classical subject. Recent work focuses on real-time applications of SLAM in distributed environments to generate and update large-scale maps. Existing SLAM solutions endow robots with capabilities to accurately estimate maps and their positions in these maps [12,13,14,15]. Yet, these solutions use representations that are not efficient. In terms of computation, many proposed algorithms either have fixed computational costs or require a complete set of sensed data. In terms of bandwidth usage, the typical point cloud representation [8,9] requires large bandwidth budgets to share with other robots. These two key shortcomings, the high computational cost (often requiring GPU acceleration) and bandwidth requirement, prohibit SLAM from being used in robots that have limited computational or memory resources, e.g., light-duty UAVs and swarm-style robots. Hence, efficient use of computational resources and communication bandwidth is critical to deploying multi-agent systems.

Recent SLAM systems also leverage deep learning frameworks to extract high-level information, such as semantics for use in downstream algorithms, e.g., natural language processing [16,17,18,19,20]. However, these systems do not leverage the semantic information to simplify the geometric representation of the sensed data or the SLAM map. This work investigates methods that promise to allow this information to feedback into low-level processing functions such as relative pose estimation and depth map sharing, or higher level tasking.

Objects with a complex pattern in the environment can be transformed into geometric primitives and can be also reconstructed from these primitives based on some visual rules, such as shape grammars [21], which can help address many of the aforementioned problems. With these modeling grammars being known by robots in advance, the robots are able to reconstruct the scene using very limited information like the model of objects (determined by semantics) and their locations with respect to the robots. Further, the grammatical description of the world makes it possible to extract abstract natural language (NL) descriptions of the world. For example, “there is a wall (associated with the shape grammars of the wall) behind a table (associated with the shape grammars of the table) at the position (x,y,z)”. This level of NL description not only permits natural-language-facilitated human–robot cooperation (NLC) but also provides a compressed description of the world. This allows closer collaboration between humans and robots which has received increasing attention in the recent decade [22]. By using NL, human intelligence at high-level task planning and robot physical capability at low-level task executions, such as force, precision, and speed, are combined to perform intuitive cooperation.

Our work takes the first step in the exploration towards the goal, to enable mobile robots to describe the world with compressed data and share maps with much less bandwidth requirement. As an initial step towards this broader goal, we limit the geometric primitives described above to planes to construct our SLAM system. We base our solution on DVO-SLAM [10] and our previous work, compute-bound and low-bandwidth RGB-D graph SLAM [23]. As a popular visual SLAM system, DVO performs well for RGB-D camera tracking and building accurate maps. It was extended in [23] with a fast plane fitting algorithm to extract surface information from scenes. A Quadtree [24] structure was also used to compress maps into a planar representation. However, this map representation is sparse and, thus, may contain large holes and ambiguous regions. Moreover, RGB-D data rather than plane data were used for odometry estimation, which lacked computation efficiency. Finally, neither RGB-D nor semantic information was available for representing the map (only geometries). In this article, we extend our previous framework for bandwidth and computation reduction with two novel depth compression algorithms and one improved independent plane fitting algorithm. These efficiently provide the system with dense plane fits. With the dense plane data available, we are able to utilize the fitted planes to calculate the odometry and then reconstruct dense maps. To provide robots with model information of the objects in the scene, we integrate a convolutional neural network (CNN) into the SLAM system to extract semantic information correlated to the planes in the environment. To summarize, the main contributions of our work are as follows:

- Two efficient and effective compression algorithms for depth images are introduced and implemented;
- A real-time fast plane fitting method is proposed which can fit planes independently of the sensor intrinsic camera perimeters;
- A real-time odometry algorithm based on plane constraints is established;
- An RGB-D SLAM approach is developed which can construct and share 3D semantic maps with much less computational cost and bandwidth requirement;
- The potential is shown to estimate semantics from compressed geometric information by feeding planes to an RGB-D CNN for semantic segmentation;

These contributions significantly advance the state of the art for RGB-D SLAM. Taking advantage of one of the geometric primitives, planes, our method provides a new representation for point clouds that can be quickly calculated and represents the data to a similar degree of geometric accuracy using far fewer parameters. The joint effect of these contributions allows agents with 3D sensing capabilities to calculate and communicate compressed map information commensurate with their onboard computational and bandwidth resources. Our results show the ability of the proposed method to compress a depth image of 1 MB in real-time to as little as 144 KB. Plane fits can also be calculated independently in real-time which enables multiple agents to efficiently share the compressed map and build the map, which helps significantly reduce the bandwidth consumption. The results of our experiments also demonstrate that by using plane data for odometry, the computational cost can be saved by as many as 12 times. Additionally, our analysis of semantic segmentation results shows the potential and benefits of extracting semantic information from compressed geometry data. As a first step towards a generalized parametric/semantic model of the world, our work motivates future research on SLAM where robots can estimate modeling information of objects from more shape primitives and share this understanding of the world with limited communication channel capacities.

The paper is organized as follows: Section 2 reviews prior work on saving computational cost and bandwidth for SLAM systems; Section 3 briefly discusses some background work that the proposed method is based on; Section 4 presents our solution; Section 5 analyzes the performance of the proposed method through extensive evaluation. Finally, Section 6 concludes the paper.

## 2. Related Work

In this section, we provide an overview of prior work done on our contributions, focusing on how bandwidth usage is reduced and how computational cost is saved by state-of-the-art SLAM systems. We also review the currently used semantic segmentation neural networks in the field, to explore the use of deep learning techniques in interpreting geometric information.

### 2.1. Bandwidth of SLAM

Due to the bandwidth constraints and limited communication range, it is challenging to share large amounts of data among the agents in a distributed SLAM system [25]. To overcome this, Montijano et al. [26] propose a distributed communication network where every robot only exchanges the local matches with its neighbors. The algorithm propagates local to global contexts to solve for a global correspondence [27,28] and manages to reduce bandwidth requirements by transmitting a subsets of the map information as a collection of sparse features. Recent research has explored the use of compact geometric representations like planes to reduce the map size [23,29,30]. These sparse representations give rise to sparse map data that may however contain large holes and ambiguous regions. Renaud et al. [31] propose a multi-agent system that reduces the required communication bandwidth and the complexity by partitioning point clouds into parts. They then compactly describe each part using discriminating features to efficiently represent the environment. Cieslewski et al. [32,33] minimize bandwidth usage by running place recognition on image frames and only sending the extracted feature vectors to the robots.

Although all of these SLAM systems work efficiently in reducing bandwidth, these solutions suffer from multiple issues. They are either not designed for RGB-D data, or they do not provide dense 3D maps as part of their SLAM solution. Moreover, they may not be optimized for distributed multi-agent systems. Our bandwidth reducing solution, in contrast, allows agents to fit planes to dense point cloud independently and to easily compare plane fits with each other. We leverage a dense planar representation of the world, as our first step to interpret the world with more geometric primitives.

The compression of sensed raw data is also critical for distributed visual-SLAM systems to perform in bandwidth constraint platforms. Shum et al. [34] present a detailed summary of image-based representations of video, textures, depth, etc. Researchers have previously tried to apply color image-based compression techniques on depth images but, considering that depth images are significantly different from color images, standard color compression techniques may not be optimal. Nevertheless, several lossy schemes which are based on color image-based compression have been proposed for compressing depth images. For example, Krishnamurthy et al. [35] use a JPEG2000 based technique to achieve an approximate compression ratio of 50× on depth images. Mehrotra et al. [36] present a lossless entropy encoding algorithm that stores the inverse depth values as integers. Wildeboer et al. [37] present an H.264 based scheme to compress depth maps from a video sequence. Pratapa et al. [38] present a random-access depth compression algorithm that generates a compressed depth image by partitioning the scene into three parts and processing each part independently.

With the lack of study in this area, in this article, we introduce and implement two novel compression algorithms for depth images. The first algorithm is an implementation of the lossless data compression library *zlib* [39] on depth data, which uses a dictionary-based compression entropy encoding scheme. The second algorithm is a novel random access compression algorithm that implements the *zlib* algorithm on 8 × 8 blocks and is described in detail in Section 4.1.

### 2.2. Computational Cost of SLAM

Another major challenge for current visual 3D SLAM approaches is to overcome the burden that processing sensed RGB-D data places on the host’s available computational resources. Centralized approaches [40,41,42] address computational cost by aggregating data from multiple robots at a central server having more computational resources where SLAM estimates can be calculated. Yet, such approaches are not viable for RGB-D data since sharing these data requires prohibitively large network bandwidth. Further, this computational model does not scale as the number of robots increases. Lajoie et al. [25] solves the SLAM optimization problem via distributed computation approaches. In this context, robots utilize only local computation and communication to optimize the SLAM pose graph and estimate robot trajectories as well as environment maps. Another distributed mapping algorithm [43] optimizes the SLAM algorithm by sharing key informative features. Recent research [44,45] has extended this implementation as a backend to larger SLAM solutions as a method to reduce the computational burden of solving multiple robot trajectories in multi-agent systems.

There has also been significant interest in compact shape models to mitigate computational issues. Planes, for example, have been used in SLAM for efficient surface data representation that can be associated and integrated with low computational complexity. Many plane-involved SLAM solutions, however, either extend feature-based SLAM with the use of planar surfaces [46], or have an orthogonal assumption on the environment which is less applicable [47]. Salas-Moreno et al. [48] build a dense planar SLAM system using a dense ICP method to estimate sensor poses, which requires GPU for real-time computation. A quaternion-based minimal plane representation is utilized by Kaess et al. [49] to update the planes during optimization without using GPU but the system does not perform well in real-time. Real-time CPU-only execution of dense planar SLAM algorithm succeeds in exceeding current popular online 3D reconstruction methods in pose estimation [29], while the computational cost can be further saved by aligning planes for loop closures instead of searching for 3D point pairs.

Although some of these solutions can be computation-efficient, they are either not applicable to 3D mapping scenarios in bandwidth-constrained contexts, or not designed for multi-agent systems. In this article, to further reduce the computational complexity, we distribute the computation burden across the SLAM system, perform plane fitting to RGB-D data in real-time, and utilize plane representation for odometry and map building.

### 2.3. Semantic Segmentation Neural Networks

Assigning meaningful semantic labels to objects on the map is a non-trivial task and there exists a great deal of prior work [50,51,52] on the subject. Some of the many approaches to this are presented here for context. The first design decision is whether to generate many labels by performing semantic segmentation or fewer with object detection and recognition. The difference is that object detection and recognition attempts to assign a label to an entire image or sub-image. Alternatively, semantic segmentation attempts to assign a label to each pixel. There are advantages and disadvantages to each approach, but in this work we are interested in making dense planar maps, so we utilize semantic segmentation.

In the context of machine learned semantic segmentation, the most well known semantic segmentation network is Mask R-CNN [53] which is a large network that is available pre-trained on a large dataset of images of everyday objects in context. Unfortunately, the objects it is trained on are poorly represented by planes and also generally make poor landmarks. Instead we use a modified version of the RedNet [54] architecture. RedNet differs from Mask R-CNN in that it was intended to be used for indoor navigation and, as such, has two branches, one for RGB images and one for depth images. These branches extract features from their respective inputs and then merge the two data streams to perform the segmentation. This has the advantage that the depth images are more useful for segmentation and extracting object edges while RGB images contain more information for object recognition. We modify the network to use plane coefficient images and retrain on the same dataset, but with the depth images pre-processed into plane coefficient images. As will be discussed in the Methodology section, operating on plane images provides an inductive bias that allows for certain classes to be more easily segmented.

### 2.4. RGB-D SLAM

We choose some representative state-of-the-art RGB-D systems and make a comparison in Table 1. Ours particularly stands out in providing bandwidth reduction and semantic information. Additionally, in contrast to [10,49,55,56], our SLAM system has frontend–backend separability. This allows the system to distribute computation tasks on different CPUs or on completely distinct connected hosts, from which a multi-agent system can benefit. Note that the discussion of bandwidth is not applicable to systems that cannot be directly deployed in a distributed network, as those systems only run on a single host. All of the mentioned SLAM systems can run in real-time according to their articles while some of them require GPU acceleration. As shown in the table, we are working on a new space in terms of bandwidth reduction and a semantic world, which makes a fair comparison difficult. The main purpose of this article is to provide different solutions to the ultimate goal which is making robots understand and communicate the knowledge of the world with limited onboard resources.

## 3. Background

In this section, we provide background on key concepts of the planar semantic SLAM which were discussed in detail in our previous publications. We will specifically focus on giving readers background on graph SLAM and the plane fitting algorithm previously described in [23].

### 3.1. Graph SLAM

Building a factor graph representation of a robot’s state is a popular technique for solving the SLAM problem [58,59,60,61,62,63,64]. Factor graphs use a sparse representation of the robot’s states to estimate the *full* trajectory of a robot and are ideal for compute-bound systems. The graph SLAM problem can be formulated as estimating the posterior probability shown in Equation (1), where a graph is constructed as the robot moves through an unknown environment map m along a trajectory represented by the sequence of random variables $x_{1:T}=\left\{x_{1},\ldots ,x_{T}\right\}$. Although moving from an initial position $x_{o}$, the robot acquires a sequence of odometry measurements $u_{1:T}=\left\{u_{1},\ldots ,u_{T}\right\}$ and perceptions of the environment $z_{1:T}=\left\{z_{1},\ldots ,z_{T}\right\}$.

(1) $$p(x_{1:T},m|u_{1:T},z_{1:T},x_{o})$$

In a graph-based SLAM approach, the robot poses are represented as nodes or vertices which relate to their position in the environment. The spatial constraint between vertices are obtained from either the odometry measurements $u_{t}$ or sensor measurements $z_{t}$ and are represented as edges. An edge constraint is obtained either between two consecutive robot positions or by aligning sensor observations between two robot locations (loop closures).

Solving the posterior leads to an optimization problem over a sum of non-linear quadratic constraints in the graph. Once the graph is constructed, we seek the configuration of the graph vertices that best satisfy the set of constraints and we seek a Gaussian approximation of the posterior distribution of Equation (1). The optimal trajectory, $x^{⋆}$, is estimated by minimizing the joint log-likelihood of all constraints as described by Equation (2) [65].

(2) $$x^{⋆}=\min_{x}\left(x_{o}^{T}\Omega_{o}x_{o}+\sum_{t}{\left[x_{t}-g\left(u_{t},x_{t-1}\right)\right]}^{T}R_{t}^{-1}\left[x_{t}-g\left(u_{t},x_{t-1}\right)\right]+\sum_{t}{\left[z_{t}-h\left(x_{t},m_{i}\right)\right]}^{T}Q_{t}^{-1}\left[z_{t}-h\left(x_{t},m_{i}\right)\right]\right)$$

The leftmost term, $x_{o}^{T}\Omega_{o}x_{o}$, represents our prior knowledge of the initial pose where $\Omega_{o}$ is the inverse covariance matrix associated with the initial motion. Often, $x_{o}$ is set to zero, anchoring the map to the origin. The middle term describes a sum over the motion constraints. The residual vector $x_{t}-g\left(u_{t},x_{t-1}\right)$, is then the difference between the expected pose, $x_{t}$, and the pose observed by applying the motion estimate $g(u_{t},x_{t-1})$. Similarly, the rightmost term describes a sum over landmark constraints. The residual vector $z_{t}-h\left(x_{t},m_{i}\right)$, is then the difference between the expected landmark pose in the global coordinate system, and the estimated global landmark pose resulting from the local landmark measurement. $R_{t}^{-1}$ and $Q_{t}^{-1}$ are the information matrices or inverse covariance matrices associated with the motion constraint and the landmark constraint, respectively.

As mentioned earlier, the minimization is commonly solved using non-linear optimization approaches like, e.g., Levenberg–Marquardt, Gauss–Newton, etc. It can also be solved efficiently by exploiting the sparse structure of the graph SLAM formulation [66], thereby allowing the use of sparse methods, such as sparse Cholesky decomposition or the method of the conjugate gradient. Many graph optimization libraries, such as the g^2^o library [67,68], leverage these sparse methods in order to quickly optimize large graphs.

Many SLAM systems can be well described in terms of two fundamental components: (1) the frontend and (2) the backend. The frontend tracks camera pose changes from RGB-D images by minimizing the reprojection errors between two image pairs, which results in a delta-pose measurement of the camera’s ego-motion and associated uncertainty [69]. The backend is responsible for large-scale map integration. The estimated camera poses and their uncertainties are used to establish a vertex obtained from keyframe data. Edges connect vertices using sensor data from connected keyframes. When two keyframes include overlapping views of the same geographic map regions, the graph creates a new constraint also called loop-closures.

One important visual SLAM implementation is direct visual odometry (DVO) [10]. DVO implements a keyframe-based approach for map building where the visual odometry algorithm uses an RGB-D pair (keyframe) and estimates odometry between this frame and subsequent frames. Once a certain threshold, such as distance, bearing change, or uncertainty, a new keyframe is established. The 3D map which is constructed on the backend is a collection of the keyframes pose at the time it was established.

Our previous work [23] extends DVO SLAM by adding several new capabilities. One of the most significant new capability allows separates the frontend, and backend components of this system into distinct applications which share information via network communication. These components can run on the same or different CPUs or on completely distinct hosts connected over a low-bandwidth network.

### 3.2. Real-Time Plane Fitting to RGB-D Data

In [23], we seek to approximate the measured depth data with 3D planar surfaces. The planar representation for 3D scene promises to significantly reduce the size of the RGB-D image data which serves to reduce both memory usage and computational costs. Plane fitting is accomplished in real-time using an ultra fast-fitting algorithm proposed in [70]. This accelerated fitting of planes is made possible by a rearrangement of the standard plane fitting error functions for RGB-D data. Standard planar representations adopt a form of equation aX+bY+cZ+d=0 where $X=Z\left(\frac{x-c_{x}}{f_{x}}\right)$ and $Y=Z\left(\frac{y-c_{y}}{f_{y}}\right)$ and Z=Z(x,y), where (x,y) is the image pixel coordinate and $\left(c_{x},c_{y},f_{x},f_{y}\right)$ are camera intrinsic parameters. We substitute the 3D reconstruction equations for the variables *X* and *Y* then simplify the resulting equation to fit directly to only the measured RGB-D depths and, in doing so, save significant computational cost.

The re-arrangement of the terms gives

(3) $$\frac{aX}{dZ}+\frac{bY}{dZ}+\frac{c}{d}+\frac{1}{Z}=0$$

In order to fit planar models to sets of 3D points, we leverage an explicit least-squares formulation to compute the coefficients of the plane as shown by the objective function in the equation. We rename the variables as follows $\alpha_{1}=\frac{a}{d}$, $\alpha_{2}=\frac{b}{d}$ and $\alpha_{3}=\frac{c}{d}$ and solve the explicit least squares fitting problem in Equation (4) below.

(4) $$f\left(x,y\right)=\sum_{\left(x,y\right)}{∥\alpha_{1}\left(\frac{x-c_{x}}{f_{x}}\right)+\alpha_{2}\left(\frac{y-c_{y}}{f_{y}}\right)+\alpha_{3}+\frac{1}{Z}∥}^{2}$$

Note that $\frac{x-c_{x}}{f_{x}}$ and $\frac{y-c_{y}}{f_{y}}$ can be pre-computed from the known integer (x,y) pixel coordinates and the calibration parameters.

This equation provides us the benefit of conceiving a computational algorithm that will fit an explicit 3D planar surface directly to the measured, i.e., perspective-projected, depth image values. The result of the fit depends on the camera intrinsic parameters $\left(f_{x},f_{y},c_{x},c_{y}\right)$. The least-squares scatter matrix for this fitting approach has the form as shown in Equation (5) below.

(5) $$M^{t}M=\sum_{i=1}^{N}\left[\begin{array}{ccc} {\left(\frac{x_{i}-c_{x}}{f_{x}}\right)}^{2} & \left(\frac{x_{i}-c_{x}}{f_{x}}\right)\left(\frac{y_{i}-c_{y}}{f_{y}}\right) & \left(\frac{x_{i}-c_{x}}{f_{x}}\right)\\ \left(\frac{x_{i}-c_{x}}{f_{x}}\right)\left(\frac{y_{i}-c_{y}}{f_{y}}\right) & {\left(\frac{y_{i}-c_{y}}{f_{y}}\right)}^{2} & \left(\frac{y_{i}-c_{y}}{f_{y}}\right)\\ \left(\frac{x_{i}-c_{x}}{f_{x}}\right) & \left(\frac{y_{i}-c_{y}}{f_{y}}\right) & 1 \end{array}\right]$$

where M denotes the matrix of planar monomials formed from the 3D (X,Y,Z) surface data having ith row $M_{i}=\left[\begin{array}{ccc} \frac{x_{i}-c_{x}}{f_{x}} & \frac{y_{i}-c_{y}}{f_{y}} & 1 \end{array}\right]$. The least squares solution for the unknown vector $\alpha^{t}=\left[\begin{array}{ccc} \alpha_{1} & \alpha_{2} & \alpha_{3} \end{array}\right]$ is then $\alpha ={\left(M^{t}M\right)}^{-1}M^{t}b$ where b is the inverse of the measured, i.e., perspective projected, depths; $b=\left[\begin{array}{ccc} \frac{1}{Z_{0}} & \ldots & \frac{1}{Z_{N}} \end{array}\right]$ which is proportional to the pixel disparity typically used for depth calculation in depth sensors [71].

It is important to note that none of the elements of the scatter matrix depend on the measured depth data and, as such, this matrix requires a constant number of operations to compute for each measured image, i.e., it can be pre-computed given the RGB-D camera parameters. Hence, explicit plane fitting in the RGB-D range space requires only computation of the vector $b=\left[\begin{array}{ccc} \frac{1}{Z_{0}} & \ldots & \frac{1}{Z_{N}} \end{array}\right]$ for each range image and the best-fit plane is given by a single matrix multiplication: ${\left(M^{t}M\right)}^{-1}M^{t}b$, where the value of $M^{t}b$ is given below:

(6) $$M^{t}b=\sum_{i=1}^{N}\frac{1}{Z_{i}}\left[\begin{array}{c} \frac{x_{i}-c_{x}}{f_{x}}\\ \frac{y_{i}-c_{y}}{f_{y}}\\ 1 \end{array}\right]$$

## 4. Methodology

The goal of the proposed approach is to efficiently and effectively compress the knowledge of the world. This enables robots with limited resources to contribute to, and potentially benefit from, 3D maps in a distributed 3D SLAM system. This goal is achieved by the following modifications to the previous work [23].

- Independent fast plane fitting—a coordinate transformation is applied so that solving the least-squares fitting problem is independent of the sensor intrinsic parameters;
- Compression algorithm—a bitmap is used to record *NaN* value locations before compressing the depth and then encode the depth with a custom dictionary-based compression algorithm;
- Odometry algorithm with planes—camera transform is calculated by aligning the compact plane fits of the point cloud instead of repetitively matching every point;
- Semantic maps—plane images is populated to an RGB-D CNN for semantic segmentation as input to show how geometries help extract semantics.

**Figure 1:**
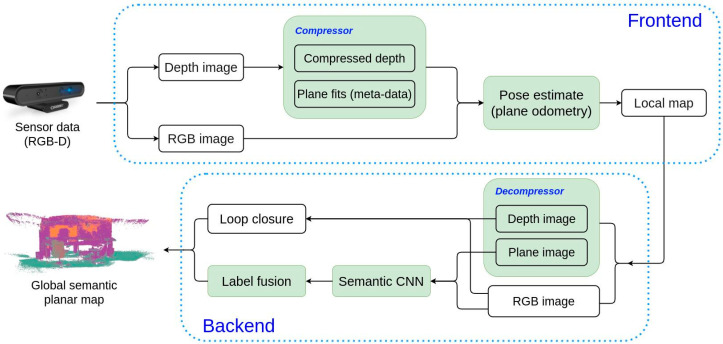
Framework of our low-bandwidth 3D planar semantic SLAM system. The frontend takes sensor data as input then performs depth compression (Section 4.1) and plane fitting (Section 4.2) in the same time. The plane coefficients and RGB images are used for fast camera pose estimation (Section 4.3). Local maps contain all image and plane data, as well as camera pose and camera parameters. The backend takes as input local maps to recover the RGB, depth, and plane images (an image of plane coefficients), and create a SLAM graph with loop closures. The semantics are predicted by a CNN and integrated into the graph to generate a global map. Note that in our experiments the loop closure detection uses depth and RGB images to perform the more accurate mapping. However, for fast loop closure detection, the depth data can be replaced with plane data.

The cumulative effect of these modifications generates a new SLAM system that advances the state-of-the-art for efficient multi-agent map building. The framework of the overall SLAM system is shown in Figure 1, and it contains two parts: (1) the frontend where the depth data are compressed and the camera pose is estimated, and (2) the backend where the global semantic planar map is generated.

### 4.1. Depth Compression

When building multi-agent distributed SLAM systems, data compression plays an important role in reducing the bandwidth budget of agents especially in visual SLAM applications that use RGB-D sensors. Although there are very well-established and highly-tuned algorithms to compress RGB data, not many algorithms are available for depth images compression. Several lossy schemes that work for standard color images have been used to compress depth images but do not perform as well. This is because the depth images have different properties than standard color images so the compression algorithms have unique needs to compensate for *NaN* values scattered through the image. This prevents typical grid-based algorithms for compression like the discrete cosine transform (DCT) in JPEG compression, or the wavelet transform in JPEG 2000. To overcome these challenges, we propose two novel depth compression techniques, (1) a *zlib* based entropy encoding technique and (2) a custom dictionary-based compression approach that allows random access.

In our first approach, we leverage *zlib* library for lossless compression of depth images. *zlib* is a free, open-source library which is used in thousands of applications for data compression like compressing TLS connections and storing Git version control files. However, to the best of our knowledge, no previous work has applied it to depth compression. *zlib* library uses a deflate-inflate method to encode and decode data which, in turn, uses a combination of the Lempel-Ziv-Storer-Szymanski (LZSS) algorithm [72] and Huffman coding [73]. The LZSS is a dictionary-based algorithm that replaces recurring bytes of data with a reference to a previously occurred byte. The algorithm uses a sliding window-based approach to find sequences of repeated data. Then the Huffman encoding breaks LZSS encoded data into blocks, and generates codes for each data block. The Huffman encoding uses a statistic-based approach to encode symbols whose lengths are based on the frequencies of occurrence. The inflate method follows similarly in reverse.

**Figure 2:**
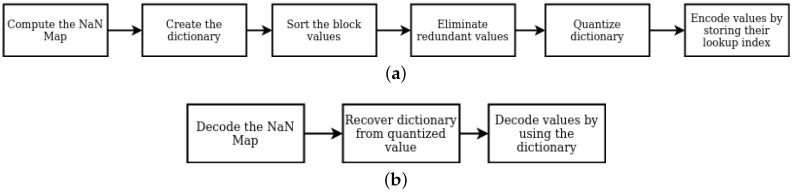
Block diagram summarizing the steps involved in (**a**) encoding and (**b**) decoding the depth map in the custom depth compression algorithm.

In our custom dictionary-based approach, we first adopt the concept of the bitmap to locate the pixels with invalid depth (*NaN* values). We then divide a 640 × 480 depth image into multiple 8 × 8 blocks. For each block, we create a bitmap to denote if an invalid pixel is existent. Specifically, in this bit pattern, for each location, if the pixel contains a *NaN* value, the bit value is set to 1. Essentially we create a bitmap representation of the block where 1 indicates where the *NaNs* are and 0 indicates where the depth measurements are. This representation requires 64 bits (8 bytes) to map the location of *NaNs*. Having all *NaN* values located, we traverse the bitmap in a zigzag pattern to convert the 2D data to a 1D vector while jumping over all of the *NaN* values that are possibly scattered in the bitmap. We then compute a compressed bitmap buffer of these *NaN* pixel locations and encode it with run-length encoding (RLE). The *non-NaN* values are encoded by a dictionary-based lossless compression algorithm using *zlib*. By filtering invalid pixels beforehand and, respectively, encode the *NaN* value locations and valid depth information, we are able to compress the geometric data while avoiding the potential problems of direct depth compression. To decompress the data, the algorithm decodes the *NaN* bitmap, and recovers the dictionary used in encoding stage to decode the values. The steps for both encoding and decoding are summarized in Figure 2.

### 4.2. Stream Meta Data

On top of our compressor, we can optionally add a layer of surface information metadata by fitting planes to 8x8 blocks of depth data. For each block of the depth image, if the block contains sufficient valid depth data (more than 50% pixels), we apply the surface fitting algorithm mentioned in Section 4.2.1 to calculate a surface representation and an entity of metadata, including the plane coefficient vector and its covariance that summarizes the log-likelihood of the pixel in that block given the plane fit.

#### 4.2.1. Independent Plane Fitting

To approximate the measured depth data with a 3D planar surface, we leverage the plane fitting algorithm discussed in Section 3.2 and extend it to serve better in multi-agent scenarios. We devise a method to fit explicit planar surfaces to local image patches without the need to know the location of the image center. This allows local N×N image patches to be compressed without the knowledge of their absolute location in the image. Let (x,y) denote the pixel coordinates within an image region bounded by upper-left coordinate $\left(x_{0},y_{0}\right)$ and lower right coordinate $\left(x_{1},y_{1}\right)$. We denote the patch center as the point $\left(x_{c},y_{c}\right)=\left(\frac{x_{1}-x_{0}}{2},\frac{y_{1}-y_{0}}{2}\right)$. We then make the coordinate transformation in Equation (7).

(7) $$\begin{array}{c} x_{i}=x_{i}^{\prime }-x_{c}+c_{x}\\ y_{i}=y_{i}^{\prime }-y_{c}+c_{y} \end{array}$$

The impact of this coordinate transformation changes the scatter matrix $M^{t}M$ from the Equation (5) to the Equation (8) in $\left(x^{\prime },y^{\prime }\right)$ shown below.

(8) $$M^{t}M=\sum_{i=1}^{N}\left[\begin{array}{ccc} \frac{{\left(x_{i}^{\prime }-x_{c}\right)}^{2}}{f_{x}^{2}} & \frac{\left(x_{i}^{\prime }-x_{c}\right)\left(y_{i}^{\prime }-y_{c}\right)}{f_{x}f_{y}} & \frac{\left(x_{i}^{\prime }-x_{c}\right)}{f_{x}}\\ \frac{\left(x_{i}^{\prime }-x_{c}\right)\left(y_{i}^{\prime }-y_{c}\right)}{f_{x}f_{y}} & \frac{{\left(y_{i}^{\prime }-y_{c}\right)}^{2}}{f_{y}^{2}} & \frac{\left(y_{i}^{\prime }-y_{c}\right)}{f_{y}}\\ \frac{\left(x_{i}^{\prime }-x_{c}\right)}{f_{x}} & \frac{\left(y_{i}^{\prime }-y_{c}\right)}{f_{y}} & 1 \end{array}\right]$$

Equations formulated in the coordinate system $\left(x^{\prime },y^{\prime }\right)$ enjoy the benefit of being independent of the sensor intrinsic camera parameters $\left(c_{x},c_{y}\right)$, and require only the knowledge of the size of the image patch, i.e., $\left(x_{0},y_{0}\right)$ and $\left(x_{1},y_{1}\right)$. Calculation of surface fits to the coordinates $\left(x^{\prime },y^{\prime }\right)$ can then be linearly put into the 3D coordinate system appropriate for a sensing camera by a single linear transformation on the estimated variable α as shown in Equation (9) below.

(9) $$T_{\alpha^{\prime }}=\left[\begin{array}{ccc} 1 & 0 & 0\\ 0 & 1 & 0\\ \frac{c_{x}-x_{c}}{f_{x}} & \frac{c_{y}-y_{c}}{f_{y}} & 1 \end{array}\right]$$

This allows inverse values for the matrix $M^{t}M$ to be pre-computed and enables the resulting fit surfaces to be placed in 3D with a single linear transformation of the estimated coefficient vector: $\alpha =T_{\alpha^{\prime }}\alpha^{\prime }$.

Various applications including multi-agent SLAM can be benefited from this separation of the plane fitting algorithm and the sensor information. In a multi-agent SLAM system where agents are likely equipped with different cameras, our camera-independent fitting algorithm allows the possibility of easily sharing and comparing plane fits across the cameras which enables an efficient cooperative SLAM system.

In addition to encoding the depth map, our compression algorithm also encodes the camera calibration information at the same time. After compressing all information, we send from frontend to backend the compressed depth image, the plane fits $\left(\frac{a}{d},\frac{b}{d},\frac{c}{d}\right)$ each of which takes three 24-bit values and plane coefficient covariance (six 24-bit values) for all blocks having 32 or more valid depths in an 8 × 8 window.

### 4.3. Plane Cloud Odometry

Similar to DVO SLAM, we use alignment results between images for odometry. Given an image pair with intensity and depth information $\left(I_{1},Z_{1}\right)$ and a second image pair taken at a later time $\left(I_{2},Z_{2}\right)$, DVO odometry algorithm seeks to estimate the set of transformation parameters $\xi_{e}$, that best aligns the images after a warp function τ(x,T) is applied to all pixels x where *T* is the transformation matrix of the camera motion and can be calculated from $\xi_{e}$ using the matrix exponential $T=\exp(\xi_{e})$. The warped location in the second image, $x^{\prime }$, of an image pixel x in the first image can be computed given the transformation matrix, *T*, and the projection function, π, from pixel coordinates to a 3D point.

(10) $$x^{\prime }=\tau \left(x,T\right)=\pi \left(T\pi^{-1}\left(x,Z_{1}\left(x\right)\right)\right)$$

The goal here is to find the best correspondence that is determined by the difference between the warped image, $W(x^{\prime })$, and the reference image, I(x) or Z(x) in terms of intensity and depth value. The error function can be written as:

(11) $$f\left(\xi \right)=\frac{1}{2}\sum_{x}{\left(W_{I}\left(x^{\prime }\right)-I\left(x\right)\right)}^{2}+\frac{1}{2}\sum_{x}{\left(W_{Z}\left(x^{\prime }\right)-Z\left(x\right)\right)}^{2}=\frac{1}{2}\sum_{x}r_{I}{\left(x\right)}^{2}+\frac{1}{2}\sum_{x}r_{Z}{\left(x\right)}^{2}$$

Note that the error term above is non-linear, and we, therefore, find ourselves in the midst of a non-linear least-squares problem. It is worth to mention that a multi-scale methodology is adopted by DVO for odometry calculation. At different levels, it first computes the residuals for intensity and depth, then computes the sum of residual weighted gradient vectors for parameter update.

The problem of matching image measurements (depth and intensity) is that it requires the existence of the same sample from the same surfaces, which is not always available with the camera moving. The common solution to this problem is to perform interpolation which introduces much computation to the problem. For example, DVO Odometry performs interpolations for depth images, intensity images, depth gradients in x,y directions, and intensity gradients in x,y directions at every iteration, as a result of which, it is very computationally expensive. This can be addressed by directly solving for the correspondences between plane fits from two plane images [30]. We use the compact representation of the world in terms of planar algebraic surfaces, i.e., surfaces having equation ax+by+cz+d=0, to establish the likelihood that a given hypothesized plane image pair can be aligned with the intent to piece together large geometric map regions in a manner similar to puzzle-solving. This is accomplished by minimizing an error function that solves for both the correspondence of planar surfaces between the plane images and the Euclidean transform that aligns these algebraic surfaces. The magnitude of the algebraic alignment error then serves as a goodness-of-fit metric to validate or refute the plane image pair hypotheses. We base our plane odometry algorithm on this and it saves much computational cost by avoiding searching for corresponding measurements from surfaces at the corresponding location. Instead, it computes the optimal alignment (in the least-squares sense) between planes that are hypothesized to have the same equation up to an unknown Euclidean transformation. For our derivation, we denote $\pi_{j}$ and $\pi_{l}$ as two collections of *N* plane equations. Equation (12) expresses the optimization problem at hand. Here we seek to estimate the transformation ${\hat{T}}_{i\to j}$ that takes the planes of $\pi_{l}$ into the coordinate system of planes $\pi_{j}$. Note that Euclidean transformations, when applied to planes, follow the transformation rule $\pi^{\prime }={\left(T^{-1}\right)}^{t}\pi$ for $\pi^{t}=\left[a,b,c,d\right]$.

(12) $${\hat{T}}_{i\to j}=\min_{T_{i\to j}}\sum_{\{i,j\}pairs}{∥\pi_{j}-{\left(T_{i,j}^{-1}\right)}^{t}\pi_{i}∥}^{2}$$

To benefit from both image measurements and algebraic representation of points, we can alternatively add a stream of metadata from our plane fitting on top of the intensity constraints in Equation (11), in replace of the depth constraints. The new error function is shown in Equation (13). We call this approach hybrid odometry. Specifically, our hybrid odometry treats plane coefficients (a,b,c,d) as four images in addition to the intensity image that is already used by DVO. The transformation to be solved needs to bring 4 plane coefficients and intensity values into an agreement between two frames. Intensity images are required to be downsampled to match the size of plane images and interpolations are performed during calculation. By taking advantage of the metadata within blocks, our method aligns images by performing calculations on a much smaller collection of values instead of visiting every pixel in the image.

(13) $$f\left(\xi \right)=\frac{1}{2}\sum_{x}r_{I}{\left(x\right)}^{2}+\frac{1}{2}\sum_{x}r_{P}{\left(x\right)}^{2}$$

We also perform graph-SLAM incorporating only motion constraints into the backend optimization like DVO SLAM. However, in contrast to DVO SLAM, our backend keyframes store compressed plane clouds in lieu of RGB-D data. This fundamentally changes the function $g(u_{t},x_{t-1})$ in the second term of the graph-SLAM optimization problem in Equation (2) from an RGB-D point cloud alignment problem to that of aligning compressed plane clouds. The new optimal pairwise motion estimate for a keyframe pair is replaced by a new odometry algorithm $g(u_{t},x_{t-1})$ that estimates the relative motion of a pair of point clouds by minimizing the corresponding pairwise plane cloud data error.

### 4.4. Semantic SLAM

Our semantic slam approach applies a CNN that takes in RGB images and plane images which are sets of plane coefficients associated with each pixel, to produce semantic labels for each input pixel. Once semantic labels are assigned to each element label fusion is used to ensure that labels are consistent across time and multiple views. These are the core components of semantic slam: semantic segmentation to generate labels and label fusion to combine and track them. These are discussed in the following subsections.

#### 4.4.1. Plane Semantic Segmentation Network (RedNet)

We use RGB + Plane coefficient data as the inputs into a multi-branch convolutional neural network that performs semantic segmentation. Semantic segmentation is a combination of image segmentation, in which pixels correspond to the same object, and object classification, identifying and labeling that object. This is useful both for producing labeled maps and for reducing the computational burden of identifying correspondences and loop closures as there is no sense in trying to match a table to a wall.

There are many networks, such as RedNet [54] and BiSeNEt [74], that use both RGB and depth information to perform semantic segmentation. These networks generally perform convolutional operations on the RGB and depth images in parallel and then fuse the information together before scaling back up to the original resolution, though implementation details differ. We are forced to retrain on a targeted dataset with modifications to the depth branch to operate on plane images.

A plane image is similar to a depth image except it contains plane coefficients instead of depth values. Since a plane can be represented with as few as 4 coefficients, we are able to reconstruct 3D geometric data into a 4 channel image but with 3D information for the following semantic segmentation work. In a real-world scene, especially an indoor scene, many of the objects can be fitted by planes, such as floor, wall, table. For this work, we operate on plane coefficients but do not merge contiguous planes so as to keep the convolutional architecture. Operating on irregular collections of planes is deferred to future work.

One advantage of performing the segmentation on planes rather than on the depth values is that depth values are independent but the plane coefficients encode information about the local geometry. Based on preliminary results, we elected to focus on modifying and retraining RedNet for our application. RedNet was originally trained on RGB+D data from the SUN RGB-D dataset [75]. We train our whole network on a modified version of the same dataset in which the depth channel has been converted to a grid of plane coefficients, i.e., plane images and the RGB images scaled to align with the plane images.

We reused the PyTorch implementation of RedNet and retrained both branches with the depth branch converted to use plane images in the place of depth images. Both branches are downsampled by necessity as calculating each plane requires at least 3 valid depth points within one fitting block. No pre-trained weights for the RGB branch are available, but we follow the training regimen specified in the original RedNet paper.

#### 4.4.2. Label Fusion

Generating a semantic map requires the temporal consistency of the semantic prediction. When observing a scene from a moving camera such as on a mobile robot, the system obtains multiple different views onto the same objects. Nevertheless, as the viewpoint varies, different semantic cues estimated by the CNN may become available and a previously semantically ambiguous region may become more distinctive. To address this problem, we perform data association by warping sequential frames of different views into a common reference view and fuse the semantics.

As discussed in Section 4.3, given a 2D image coordinate $x\in R^{2}$, the warped pixel location can be determined by Equation (10). With the warped pixel location, we can find the pixel correspondences for two label images $S_{A}$, $S_{B}$ in sequential keyframes sharing a common field of view (FOV). We then compare the labels and their associated prediction confidence for those pixels in the common FOV. If the labels are the same for two corresponded pixels, we remain the same label in the label image of the second view and update the predicted confidence by averaging two confidence. If not, we update the pixel label in both label images with the label of higher confidence. The new confidence is obtained by lowering down the higher confidence by 10% as a penalty to semantic inconsistency.

The underlying intuition of our label fusion is that corresponding pixels must have the same semantic label, as well as similar (but not necessarily the same) prediction confidence. Unlike the photo-consistency assumption adopted by tracking algorithms like DVO SLAM, the semantic consistency assumption is comparatively weak since it is not anchored to any actual measurement. However, it is possible to use it as a constraint for graph optimization. In this work, we only focus on using label fusion to generate a global semantic map, not for any optimization.

## 5. Experiments and Results

In this section, we evaluate the key components of our SLAM system that we contribute to. We conduct experiments on each component separately and compare it to the state of the art when available. Specifically, (1) we compare and analyze the size of our compression data to the raw data, showing that bandwidth reduction on sharing the geometry information among agents. (2) We also compare our odometry algorithms with a popular RGB-D camera tracking algorithm DVO odometry, demonstrating that using plane data accelerates the odometry estimation process by reducing the data size and calculation complexity. (3) We then evaluate the performance of our CNN with a common RGB-D semantic segmentation CNN, RedNet. Our results outperforming RedNet on some classes suggests the potential of extracting semantics from plane data in an indoor scene where most objects have regular shapes. The TUM RGB-D dataset [76] is used for evaluation and comparison for most of the experiments while the neural network training uses the SUN-RGBD dataset. More details are discussed in the following sections, respectively.

### 5.1. Depth Compression

**Figure 3:**
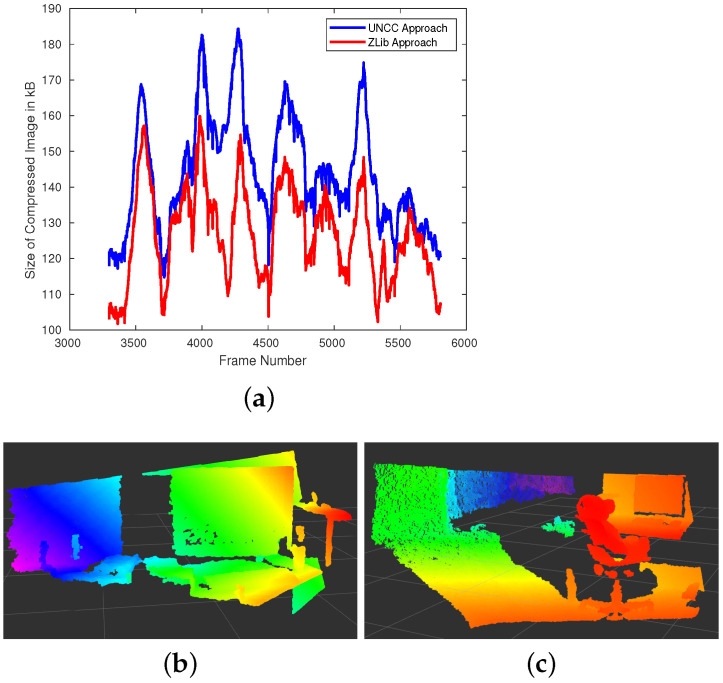
(**a**) The size of the compressed depth image (in kB) for each frame in depth dataset. (**b**) The depth point cloud capture at frame 4000. The depth image consists of objects near the camera thus containing more information to encode (as seen on the plot). (**c**) The depth point cloud at frame 4500. The majority of the objects captured are relatively far from the sensor, resulting in a low-resolution depth image thus resulting in a smaller compressed depth image.

Depth compression for SLAM is a relatively new concept and to the best of our knowledge, there exists no open-source implementation of compression on non-synthetic depth data. In this work, we present two novel depth compression techniques, *zlib* Compression and the University of North Carolina at Charlotte (UNCC) compression technique. We conduct our experiments on a laptop with an Intel Core i9 processor (no GPU used). We evaluate the performance of the two algorithms by examining the time taken to encode and decode a depth image and by investigating the size of the compressed depth map. Figure 3a shows the size of the compressed depth map for each of the 2510 depth images from a benchmark TUM RGB-D dataset. Table 2 provides statistics on the size of the compressed depth images for the same experiment. It is observed that both the *zlib* and UNCC Compression have a compression ratio of 9.6× and 8.5×, respectively. As expected, the UNCC compression algorithm has a larger compressed image, as it encodes the compressed image with meta-data containing the plane coefficients. Additionally, each 8×8 block has its own dictionary taking up more space.

As described in the previous section, the UNCC compression algorithm splits an image into 8×8 blocks and encode the non-*NaN* values using a dictionary generated by the *zlib* algorithm, which means that each 8×8 block has a dictionary that needs to be encoded. This gives the user flexibility in the decoding process in terms of decoding only parts of the image and parallelizing the decoding process. In addition to the depth map, the UNCC compression technique also encodes the camera calibration information and the plane fits for each block, thus taking up more time and size to encode a depth map. Upon further investigation, the average size difference for the encoded depth map produced by the *zlib* algorithm and the UNCC compression is ∼17 kB.

Figure 3a shows the size of the compressed depth image obtained from both the *zlib* and UNCC algorithms for each frame in the test dataset. Depth sensors perform better when objects are near the camera when compared to objects that are further away. This means that they encode more information on nearby objects producing high-resolution maps on scenes with lots of objects near the cameras like the one shown in Figure 3b as compared to the scene shown in Figure 3c. As the amount of information encoded in the original depth image is high, it results in a larger compressed image, which is seen in Figure 3a. Figure 3b,c are the 4000th and 4500th frames in the dataset, respectively.

We show the statistics for the time taken to encode and decode depth images for the two compression algorithms in Table 3. The time taken to decode the compressed depth image is significantly lower than the time taken to encode the data, especially for the UNCC compression technique. This is expected because to encode the depth map, the algorithm has to sort values in an 8 × 8 block and then remove redundant values to generate the dictionary, which is not necessary for decoding. The small duration to decode the depth image and the small size of the compressed image opens the door to storing the depth image in a compressed format. This frees up storage space for running the algorithm for longer and saving more data in memory.

In addition, the ability of the UNCC algorithm decoding blocks individually allows reconstruction of the depth map by sharing only the plane metadata between the frontend and the backend. It is noted that it takes an average of 1.24 μs to decode a single block from the compressed depth image which is comparable to Pratapa et al. [38] who present a random access compression technique on individual depth frames. This allows the possibility to further decrease the bandwidth of the computation load on both the frontend and the backend in future work.

A key difference between UNCC compression technique and the *zlib* technique is that the UNCC technique allows the user to change the behavior of the compressed depth image by varying the bits per symbol (BPS) and the quantization step size (QSS). The BPS determines the number of bits in each symbol of the dictionary whereas QSS determines how close two neighboring entries in a dictionary can be. The QSS values are represented as the inverse of the distance threshold between depth entries and have the units of m${}^{-1}$.

**Figure 4:**
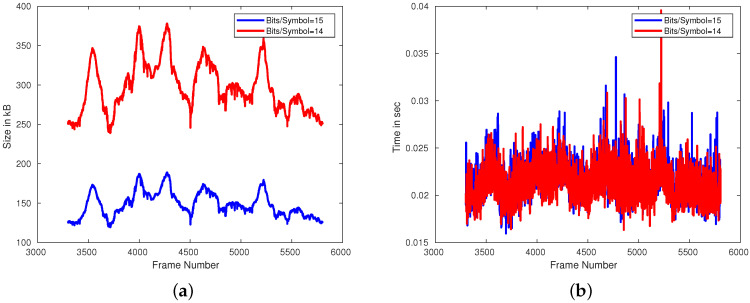
Plot showing (**a**) the changes in the size of the compressed depth image and (**b**) the total time take to encode and decode a depth image when the bits per symbol is varied.

Figure 4 shows the variations in the size of the compressed depth image and the total time taken to encode and decode a depth image for every frame in the dataset. We note that while the size of the encoded depth image changes significantly, the time taken to encode and decode the depth image does not change. Upon further investigation, we find that the majority of the time taken to perform the encoding operation is taken up by the bit packing algorithms and the time taken to move data to and from memory. Hence, we notice no significant changes in time when both BPS and QSS parameters are varied. Table 4 summarizes the size and time when the parameters are varied.

### 5.2. Odometry Estimation

**Figure 5:**
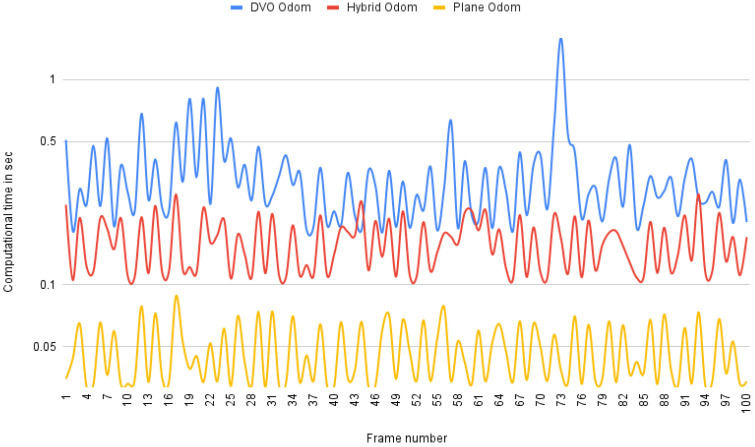
Average computational time of different odometry algorithms.

Odometry can be estimated using plane coefficients much faster than using depth and intensity measurements as DVO odometry does. To the best of our knowledge, popular RGB-D SLAM systems perform very similarly in odometry estimation to DVO odometry. Instead of matching depth for different frames, we match the algebraic representation of the surface itself. As discussed in Section 4.3, plane odometry, achieved by aligning two surface representation, avoids the effort of looking for corresponding measurements from surfaces at the corresponding locations. All of the points that are on the same surface would share the same plane coefficients, making the plane representation of the point cloud much more efficient. This is confirmed by our experiment results. We compare our plane odometry to our implementation of DVO odometry in Matlab and measure the computation time for odometry estimation. It is worth noting that DVO does an excellent job in architecture acceleration and is able to achieve real-time odometry computation. Our implementation of it is not metrically consistent with the wall-clock analysis of running their algorithm. Additionally, unlike the original DVO implementation with multi-scale calculation on the images, we only compute odometry at one single scale as the depth images and plane images have different sizes, which may require different scale numbers. Our experiments are conducted on the benchmark TUM RGB-D dataset. The results of our evaluation on 100 pairs of frames are presented in Figure 5. It shows that our plane odometry is far more computationally efficient, running at about 12 times faster overall than DVO odometry.

To take advantage of both image measurement and algebraic representation, as proposed in Section 4.3 we integrate our plane representation into DVO odometry. In this hybrid odometry approach, by grouping depth data into 8×8 blocks and representing them with 4 coefficients (a,b,c,d), more computation savings can be achieved as visiting over all pixels can be avoided (although it actually introduces more interpolation operations). The experiment results are also included in Figure 5. Our hybrid odometry performs between DVO and plane odometry in terms of computational cost. It overall runs 3 times faster than DVO and 4 times slower than plane odometry.

The acceleration of odometry calculation compared to DVO odometry, a real-time implementation, suggests the real-time capability of our algorithms. Given that RGB-D image data are captured at a resolution of N=640×480≈307k and a frame rate of 30 images/second, these computational savings from using planar representation for odometry calculation may significantly affect the run-time of real-time image processing algorithms for this class of image sensors.

We also compare the accuracy of estimated odometry from the three aforementioned algorithms with ground truth and are shown in Table 5. Although benefiting from handling much fewer data to achieve higher processing speed, both hybrid odometry and plane odometry suffer from larger error and variance than DVO odometry. This provides an insight to balance the trade-off here based on the real application, for example, to achieve a good balance between speed and accuracy performance, in a multi-scale odometry calculation scenario, plane odometry can be applied in the higher level to initialize the estimator weights for lower-level DVO odometry to leverage.

### 5.3. Semantic Segmentation

#### 5.3.1. Network Training

We trained our modified version of RedNet architecture in PyTorch with a ResNet34 backbone from random initialization on the preprocessed SUN RGB-D dataset in which the depth channel has been converted to a grid of plane coefficients. The SUN RGB-D dataset contains a vocabulary of 37 classes from indoor scenes. The optimizer used was stochastic gradient descent with a learning rate of 0.002, the momentum of 0.9, and a weight decay rate of 0.0001. The training ran for 250 epochs on four NVIDIA 1080 GPUs.

#### 5.3.2. Performance Evaluation

**Figure 6:**
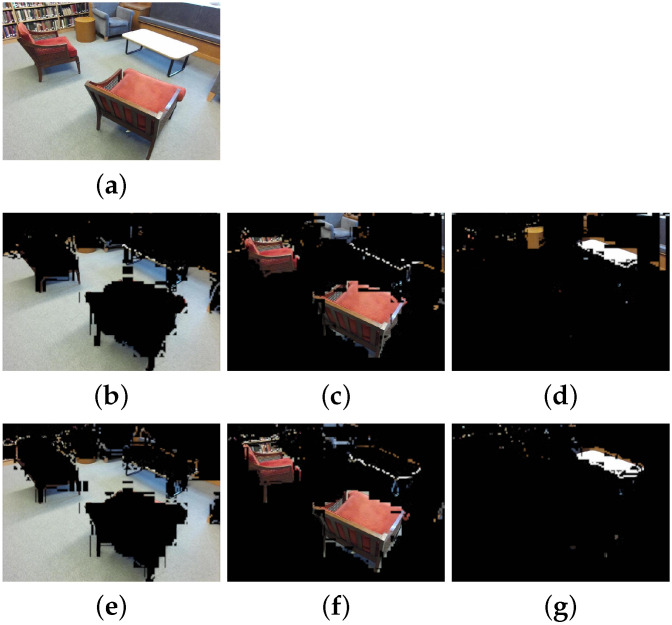
Qualitative test set results. (**a**) an example RGB image. (**b**–**d**) masked images for segmentation ground truth of three classes (floor, chair, table) respectively. (**e**–**g**) corresponding masked images for the segmentation predictions by our network.

We evaluate our modified version of RedNet architecture on the test dataset from the preprocessed SUN RGB-D dataset. Overall, our network performs well on the modified SUN RGB-D dataset described above but compares unfavorably to the original implementation. This is partially attributable to the downsampling required to calculate the plane coefficients, in this case shrinking the images by a factor of roughly four, and to the architecture not being structured to fully exploit the information in the plane coefficients. As a result, we achieved an overall pixel accuracy of 62.15% as opposed to 80.8% for the original RedNet and a mIoU of 0.272 as opposed to 0.468. The qualitative performance, shown in Figure 6, indicates it is fit for the purpose to generate semantic labels for mapping purposes. Please note that both the ground truth (top row) and predicted (bottom row) show artifacts from repeated down/upsampling and lossy compression used solely for exporting the data to make this figure.

Those top-line statistics do not tell the whole story though. Table 6 shows the mean intersection over union (mIoU) for each of the SUN RGB-D classes. The mIoU values show that performance is much better on common classes that are well represented in the training data and classes that are well represented by large planes. Ceilings, walls, and floors have the highest mean pixel accuracy and mIoU, as is to be expected. Other classes such as toilets and sink have unexpectedly high scores, presumably because they are isolated in view and protrude from walls and floors. For the top ten classes as ranked by mIoU, the accuracy for just those classes was calculated and listed in the third column. For the top three, the mean pixel accuracy is even higher than the overall accuracy of 81.3% reported for the original implementation of RedNet based on the larger ResNet-50 backbone and trained for longer on RGB-D data.

#### 5.3.3. Semantic Map

**Figure 7:**
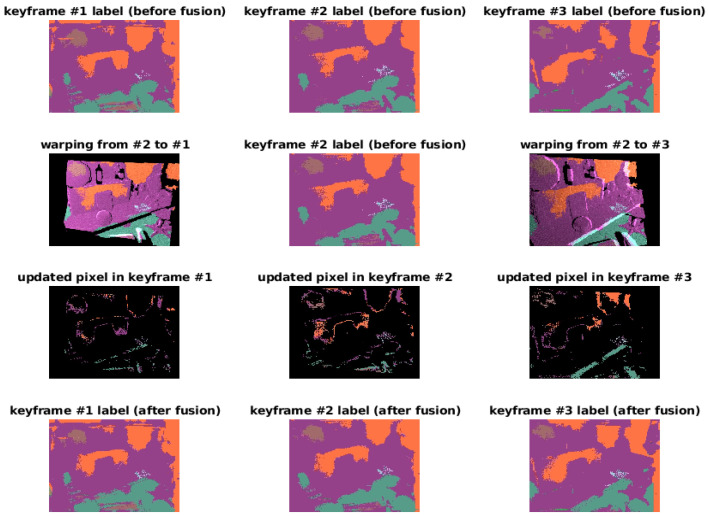
Label fusion: the first row shows three keyframe label images. The second row shows the reprojection of the image in the middle to the keyframe to the previous and next keyframe using the odometry measurement. The third row shows the pixels where the labels are updated by the fusion and the last row shows the results of label fusion. Labels across three keyframes are consistent after fusion.

We show an example of our label fusion results in Figure 7. By performing the warping we can align different keyframes if an overlap exists (second row). Label fusion will update the labels based on prediction confidence for all of the keyframe labeling results. From the last row of Figure 7, we can observe the consistency of labeling in different keyframes, for example, after performing fusion many pixels of the chair in the right image are modified and is consistent with the left image. Such consistency does not appear in the keyframes before the fusion (first row). Note that this result is only for the purpose of display the process of the label fusion. The labeling accuracy in terms of labels and associated confidence are dependent on both the network and the data we run experiments on, i.e., if the bagfile we use contains enough objects that the network can recognize after training. This is not the focus of the discussion.

**Figure 8:**
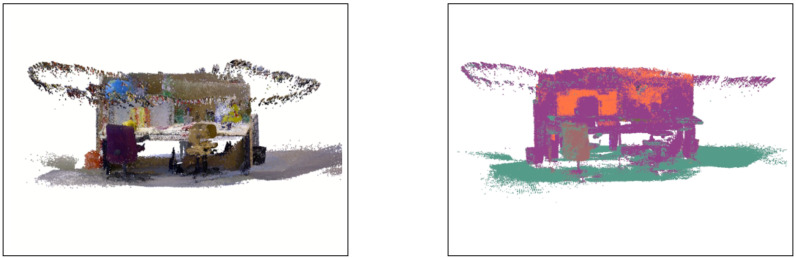
Our global map with RGB appearance (**left**) and semantic map (**right**). The camera trajectories are also shown in the map encircling the office scene.

We also present an example of our planar map with textures, as well as with semantic labels in Figure 8. Note that these maps are represented by plane clouds which are a collection of smaller planar blocks instead of point clouds. The bagfile we use for experiments does not contain sufficient objects that can be recognized by our trained network, thus the label shown there are not necessarily perfectly reliable compared to the ground truth.

## 6. Conclusions

In this article, we propose a semantic SLAM system that uses planar surfaces to reduce the resources for communicating the complexity of the world. We propose two depth compression algorithms and integrate our plane fitting algorithms on top of them. The plane fits can be computed efficiently and independently of the sensor intrinsic camera parameters, and these planes can be used for many purposes, such as fast odometry estimation. We also extend maps with semantic information predicted from sparse geometries by a CNN. Although the CNN architecture we adopt is not designated for the plane data and to be able to use it to train on plane data, we treat planar information as regular images, it still shows the potential to match semantic labels with planar models of the objects for efficient mapping. More research can be performed to address the aforementioned problems. With semantics and shape models available, our approach enables us to share among the robots very compressed knowledge of the world. In the future, we can explore more complicated geometry primitives for object representation. Estimating semantics and modeling rules at the same time is another interesting topic. The ultimate goal of this work is to enable robots to achieve high-level tasking efficiently in distributed and collaborative settings.

## Figures and Tables

**Table 1:** Comparison of selected existing RGB-D SLAM with our SLAM system.

	Frontend–Backend Separability	Bandwidth Reduction	CPU Only	Local Mapping	Semantic
DVO [10]		-	✓	Point-Based	
ORB-SLAM2 [9]		-	✓	Point-Based	
Bundle Fusion [55]		-		Volumetric	
BAD-SLAM [56]	✓			Point-Based	
Dense Planar SLAM [48]		-		Plane-Based	
SLAM with Infinite Planes [49]		-	✓	Plane-Based	
Point-Plane SLAM [57]	✓		✓	Plane-Based	
**Ours**	✓	✓	✓	Plane-Based	✓

**Table 2:** Table summarizing the compressed image size statistics.

Compression	*zlib* Compression	UNCC Compression	Original Size
Mean [kB]	127.16	144.24	1228.8
Std Dev. [kB]	12.99	15.74	N/A
Max [kB]	159.84	184.32	N/A
Min [kB]	101.72	114.76	N/A

**Table 3:** Statistics for the time taken to encode and decode depth images for the two compression algorithms.

Compression Algorithm	*zlib* Compression			UNCC Compression		
	Encode	Decode	Total	Encode	Decode	Total
Mean [μs]	5544.89	3844.98	9389.87	13,997	1665.93	15,663.26
Std. Dev. [μs]	433.74	191.97	601.79	818.75	62.68	869.15
Max [μs]	9568	5691	15,259	22,485	2930	24,524
Min [μs]	4590	3312	7944	12,044	1505	13,592

**Table 4:** Average size of compressed image and total time while varying QSS and BPS.

Scenario	Avg. Size of Compressed Image (kB)	Total Time (s)
Quantization Step Size = 1000 Bits/Symbol = 15	144.24	0.015
Quantization Step Size = 700 Bits/Symbol = 15	446.81	0.02
Quantization Step Size = 1000 Bits/Symbol = 14	297.87	0.021

**Table 5:** Accuracy performance of different odometry algorithms (yaw, pitch, and roll are in degree, and tx, ty, and tz are in meters).

	Yaw (${}^{\circ }$)	Pitch (${}^{\circ }$)	Roll (${}^{\circ }$)	tx (m)	ty (m)	tz (m)
RMSE (DVO)	0.2741	03646	0.2042	0.0070	0.0028	0.0049
Std. Dev (DVO)	0.3585	0.4443	0.2969	0.0051	0.0042	0.0071
RMSE (Hybrid)	0.9417	1.7444	1.9219	0.0430	0.0441	0.0487
Std. Dev (Hybrid)	2.9798	5.9445	5.2683	0.1842	0.1830	0.1485
RMSE (Plane)	1.7984	1.9692	0.4452	0.0552	0.0489	0.1126
Std. Dev (Plane)	1.2027	1.0511	0.5727	0.0687	0.0312	0.0412

**Table 6:** Mean intersection over Union for each of the SUN RGB-D classes.

Class	mIoU	Acc.	Class	mIoU	Class	mIoU	Class	mIoU
Floor	0.766	0.941	Sofa	0.366	Whiteboard	0.235	Box	0.1115
Ceiling	0.547	0.832	Window	0.358	Counter	0.208	Person	0.1081
Wall	0.519	0.910	Mirror	0.340	Bookshelf	0.197	Night Stand	0.0985
Chair	0.502	0.810	Cabinet	0.318	Lamp	0.187	Bag	0.0680
Curtain	0.445	0.598	Door	0.296	Blinds	0.182	Shelves	0.05767
Toilet	0.437	0.522	Fridge	0.294	Bookshelf	0.164	Shower Curtain	0.0051
Sink	0.421	0.623	Dresser	0.286	Desk	0.139	Floor Mat	0.0
Table	0.120	0.642	TV	0.279	Clothes	0.130	-	-
Bed	0.412	0.711	Pillow	0.254	Towel	0.127	-	-
Bathtub	0.411	0.481	Picture	0.250	Paper	0.121	-	-

## References

[1] Colas F., Mahesh S., Pomerleau F., Liu M., Siegwart R. 3D path planning and execution for search and rescue ground robots. Proceedings of the 2013 IEEE/RSJ International Conference on Intelligent Robots and Systems.

[2] Pathak K., Birk A., Schwertfeger S., Delchef I., Markov S. Fully autonomous operations of a jacobs rugbot in the robocup rescue robot league 2006. Proceedings of the 2007 IEEE International Workshop on Safety, Security and Rescue Robotics.

[3] Thrun S., Thayer S., Whittaker W., Baker C., Burgard W., Ferguson D., Hahnel D., Montemerlo D., Morris A., Omohundro Z. (2004). Autonomous exploration and mapping of abandoned mines. IEEE Robot. Autom. Mag..

[4] Grisetti G., Stachniss C., Burgard W. (2007). Improved techniques for grid mapping with rao-blackwellized particle filters. IEEE Trans. Robot..

[5] Birk A., Vaskevicius N., Pathak K., Schwertfeger S., Poppinga J., Buelow H. (2009). 3-D perception and modeling. IEEE Robot. Autom. Mag..

[6] Kohlbrecher S., Von Stryk O., Meyer J., Klingauf U. A flexible and scalable SLAM system with full 3D motion estimation. Proceedings of the 2011 IEEE International Symposium on Safety, Security, and Rescue Robotics.

[7] Civera J., Lee S.H. (2019). RGB-D Odometry and SLAM. RGB-D Image Analysis and Processing.

[8] Engel J., Schöps T., Cremers D. (2014). LSD-SLAM: Large-Scale Direct Monocular SLAM. Proceedings of the European Conference on Computer Vision (ECCV).

[9] Mur-Artal R., Tardos J.D. (2017). ORB-SLAM2: An Open-Source SLAM System for Monocular, Stereo, and RGB-D Cameras. IEEE Trans. Robot..

[10] Kerl C., Sturm J., Cremers D. Dense visual SLAM for RGB-D cameras. Proceedings of the 2013 IEEE/RSJ International Conference on Intelligent Robots and Systems.

[11] Wang C., Yuan J., Xie L. Non-iterative SLAM. Proceedings of the 2017 18th International Conference on Advanced Robotics (ICAR).

[12] Kähler O., Prisacariu V.A., Murray D.W. (2016). Real-Time Large-Scale Dense 3D Reconstruction with Loop Closure. European Conference on Computer Vision(ECCV).

[13] Whelan T., Kaess M., Johannsson H., Fallon M., Leonard J.J., McDonald J. (2015). Real-time large scale dense RGB-D SLAM with volumetric fusion. Int. J. Robot. Res. Spec. Issue Robot Vis..

[14] Rünz M., Agapito L. Co-Fusion: Real-time Segmentation, Tracking and Fusion of Multiple Objects. Proceedings of the 2017 IEEE International Conference on Robotics and Automation (ICRA).

[15] Whelan T., Salas-Moreno R.F., Glocker B., Davison A.J., Leutenegger S. (2016). ElasticFusion: Real-time dense SLAM and light source estimation. Int. J. Robot. Res..

[16] McCormac J., Handa A., Davison A., Leutenegger S. SemanticFusion: Dense 3D semantic mapping with convolutional neural networks. Proceedings of the 2017 IEEE International Conference on Robotics and Automation (ICRA).

[17] Dubé R., Cramariuc A., Dugas D., Sommer H., Dymczyk M., Nieto J., Siegwart R., Cadena C. (2020). SegMap: Segment-based mapping and localization using data-driven descriptors. Int. J. Robot. Res..

[18] Yang S., Huang Y., Scherer S. Semantic 3D occupancy mapping through efficient high order CRFs. Proceedings of the 2017 IEEE/RSJ International Conference on Intelligent Robots and Systems (IROS).

[19] Li X., Belaroussi R. (2016). Semi-dense 3d semantic mapping from monocular slam. arXiv.

[20] Xuan Z., David F. (2018). Real-Time Voxel Based 3D Semantic Mapping with a Hand Held RGB-D Camera. https://github.com/floatlazer/semantic_slam.

[21] Willis A.R., Ganesh P., Volle K., Zhang J., Brink K. (2021). Volumetric procedural models for shape representation. Graph. Vis. Comput..

[22] Liu R., Zhang X. (2019). A review of methodologies for natural-language-facilitated human–robot cooperation. Int. J. Adv. Robot. Syst..

[23] Zhang J., Willis A.R., Godwin J. Compute-bound and low-bandwidth distributed 3D graph-SLAM. Proceedings of the Unmanned Systems Technology XXII.

[24] Finkel R. (1974). Quad Trees: A Data Structure for Retrieval on Composite Keys. Acta Inf..

[25] Lajoie P.Y., Ramtoula B., Chang Y., Carlone L., Beltrame G. (2019). DOOR-SLAM: Distributed, Online, and Outlier Resilient SLAM for Robotic Teams. arXiv.

[26] Montijano E., Aragues R., Sagüés C. (2013). Distributed data association in robotic networks with cameras and limited communications. IEEE Trans. Robot..

[27] Nettleton E., Thrun S., Durrant-Whyte H., Sukkarieh S. (2003). Decentralised SLAM with low-bandwidth communication for teams of vehicles. Field and Service Robotics.

[28] Tardioli D., Montijano E., Mosteo A.R. Visual data association in narrow-bandwidth networks. Proceedings of the 2015 IEEE/RSJ International Conference on Intelligent Robots and Systems (IROS).

[29] Hsiao M., Westman E., Zhang G., Kaess M. Keyframe-based dense planar SLAM. Proceedings of the 2017 IEEE International Conference on Robotics and Automation (ICRA).

[30] Brink K.M., Zhang J., Willis A.R., Sherrill R.E., Godwin J.L. Maplets: An Efficient Approach for Cooperative SLAM Map Building Under Communication and Computation Constraints. Proceedings of the IEEE/ION Position Location and Navigation Symposium.

[31] Dubé R., Gawel A., Sommer H., Nieto J., Siegwart R., Cadena C. An online multi-robot SLAM system for 3d lidars. Proceedings of the 2017 IEEE/RSJ International Conference on Intelligent Robots and Systems (IROS).

[32] Cieslewski T., Scaramuzza D. (2017). Efficient decentralized visual place recognition using a distributed inverted index. IEEE Robot. Autom. Lett..

[33] Cieslewski T., Scaramuzza D. Efficient decentralized visual place recognition from full-image descriptors. Proceedings of the 2017 International Symposium on Multi-Robot and Multi-Agent Systems (MRS).

[34] Shum H.Y., Kang S.B., Chan S.C. (2003). Survey of image-based representations and compression techniques. IEEE Trans. Circuits Syst. Video Technol..

[35] Krishnamurthy R., Chai B.B., Tao H., Sethuraman S. Compression and transmission of depth maps for image-based rendering. Proceedings of the 2001 International Conference on Image Processing (Cat. No. 01CH37205).

[36] Mehrotra S., Zhang Z., Cai Q., Zhang C., Chou P.A. Low-complexity, near-lossless coding of depth maps from kinect-like depth cameras. Proceedings of the 2011 IEEE 13th International Workshop on Multimedia Signal Processing.

[37] Wildeboer M.O., Yendo T., Tehrani M.P., Fujii T., Tanimoto M. Color based depth up-sampling for depth compression. Proceedings of the 28th Picture Coding Symposium.

[38] Pratapa S., Manocha D. RANDM: Random Access Depth Map Compression Using Range-Partitioning and Global Dictionary. Proceedings of the Symposium on Interactive 3D Graphics and Games.

[39] zlib. https://zlib.net.

[40] Bailey T., Bryson M., Mu H., Vial J., McCalman L., Durrant-Whyte H. Decentralised cooperative localisation for heterogeneous teams of mobile robots. Proceedings of the 2011 IEEE International Conference on Robotics and Automation.

[41] Lazaro M.T., Paz L.M., Pinies P., Castellanos J.A., Grisetti G. Multi-robot SLAM using condensed measurements. Proceedings of the 2013 IEEE/RSJ International Conference on Intelligent Robots and Systems.

[42] Dong J., Nelson E., Indelman V., Michael N., Dellaert F. Distributed real-time cooperative localization and mapping using an uncertainty-aware expectation maximization approach. Proceedings of the 2015 IEEE International Conference on Robotics and Automation (ICRA).

[43] Choudhary S., Carlone L., Nieto C., Rogers J., Christensen H.I., Dellaert F. (2017). Distributed mapping with privacy and communication constraints: Lightweight algorithms and object-based models. Int. J. Robot. Res..

[44] Cieslewski T., Choudhary S., Scaramuzza D. Data-efficient decentralized visual SLAM. Proceedings of the 2018 IEEE International Conference on Robotics and Automation (ICRA).

[45] Wang W., Jadhav N., Vohs P., Hughes N., Mazumder M., Gil S. (2019). Active Rendezvous for Multi-Robot Pose Graph Optimization Using Sensing Over Wi-Fi. https://arxiv.org/pdf/1907.05538.pdf.

[46] Trevor A., Rogers J., Christensen H. Planar surface SLAM with 3D and 2D sensors. Proceedings of the 2012 IEEE International Conference on Robotics and Automation (ICRA).

[47] Nguyen V., Harati A., Martinelli A., Tomatis N., Sa B. Orthogonal SLAM: A step toward lightweight indoor autonomous navigation. Proceedings of the IEEE/RSJ Intenational Conference on Intelligent Robots and Systems, IROS.

[48] Salas-Moreno R.F., Glocken B., Kelly P.H.J., Davison A.J. Dense planar SLAM. Proceedings of the 2014 IEEE International Symposium on Mixed and Augmented Reality (ISMAR).

[49] Kaess M. Simultaneous localization and mapping with infinite planes. Proceedings of the 2015 IEEE International Conference on Robotics and Automation (ICRA).

[50] Simonyan K., Zisserman A. (2014). Very deep convolutional networks for large-scale image recognition. arXiv.

[51] Iandola F., Moskewicz M., Karayev S., Girshick R., Darrell T., Keutzer K. (2014). Densenet: Implementing efficient convnet descriptor pyramids. arXiv.

[52] Howard A.G., Zhu M., Chen B., Kalenichenko D., Wang W., Weyand T., Andreetto M., Adam H. (2017). Mobilenets: Efficient convolutional neural networks for mobile vision applications. arXiv.

[53] He K., Gkioxari G., Dollár P., Girshick R. Mask r-cnn. Proceedings of the IEEE international Conference on Computer Vision.

[54] Jiang J., Zheng L., Luo F., Zhang Z. (2018). Rednet: Residual encoder-decoder network for indoor rgb-d semantic segmentation. arXiv.

[55] Dai A., Nießner M., Zollhöfer M., Izadi S., Theobalt C. (2017). BundleFusion. ACM Trans. Graph..

[56] Schops T., Sattler T., Pollefeys M. Bad slam: Bundle adjusted direct rgb-d slam. Proceedings of the IEEE/CVF Conference on Computer Vision and Pattern Recognition.

[57] Zhang X., Wang W., Qi X., Liao Z., Wei R. (2019). Point-Plane SLAM Using Supposed Planes for Indoor Environments. Sensors.

[58] Lu F., Milios E. (1997). Globally consistent range scan alignment for environment mapping. Auton. Robot..

[59] Thrun S., Montemerlo M. (2006). The graph SLAM algorithm with applications to large-scale mapping of urban structures. Int. J. Robot. Res..

[60] Dellaert F., Kaess M. (2006). Square Root SAM: Simultaneous localization and mapping via square root information smoothing. Int. J. Robot. Res..

[61] Olson E., Leonard J., Teller S. Fast iterative alignment of pose graphs with poor initial estimates. Proceedings of the 2006 IEEE International Conference on Robotics and Automation, ICRA.

[62] Eustice R.M., Singh H., Leonard J.J. (2006). Exactly sparse delayed-state filters for view-based SLAM. IEEE Trans. Robot..

[63] Konolige K., Agrawal M. (2008). FrameSLAM: From bundle adjustment to real-time visual mapping. IEEE Trans. Robot..

[64] Wheeler D.O., Koch D.P., Jackson J.S., McLain T.W., Beard R.W. (2018). Relative navigation: A keyframe-based approach for observable GPS-degraded navigation. IEEE Control Syst. Mag..

[65] Thrun S., Burgard W., Fox D. (2005). Probabilistic Robotics (Intelligent Robotics and Autonomous Agents).

[66] Grisetti G., Kummerle R., Stachniss C., Burgard W. (2010). A Tutorial on Graph-Based SLAM. IEEE Intell. Transp. Syst. Mag..

[67] Kümmerle R., Grisetti G., Strasdat H., Konolige K., Burgard W. G^2^o: A general framework for graph optimization. Proceedings of the 2011 IEEE International Conference on Robotics and Automation.

[68] Dellaert F. (2012). Factor Graphs and GTSAM: A Hands-On Introduction.

[69] Ganesh P., Volle K., Willis A.R., Brink K.M. Three Flavors of RGB-D Visual Odometry: Analysis of cost function compromises and covariance estimation accuracy. Proceedings of the 2020 IEEE/ION Position, Location and Navigation Symposium (PLANS).

[70] Papadakis J., Willis A.R. Real-time surface fitting to RGBD sensor data. Proceedings of the SoutheastCon.

[71] Khoshelham K., Elberink S.O. (2012). Accuracy and resolution of kinect depth data for indoor mapping applications. Sensors.

[72] Storer J.A., Szymanski T.G. (1982). Data compression via textual substitution. JACM.

[73] Huffman D.A. (1952). A method for the construction of minimum-redundancy codes. Proc. IRE.

[74] Yu C., Wang J., Peng C., Gao C., Yu G., Sang N. Bisenet: Bilateral segmentation network for real-time semantic segmentation. Proceedings of the European Conference on Computer Vision (ECCV).

[75] Song S., Lichtenberg S.P., Xiao J. Sun rgb-d: A rgb-d scene understanding benchmark suite. Proceedings of the IEEE Conference on Computer Vision and Pattern Recognition.

[76] Sturm J., Engelhard N., Endres F., Burgard W., Cremers D. A benchmark for the evaluation of RGB-D SLAM systems. Proceedings of the 2012 IEEE/RSJ International Conference on Intelligent Robots and Systems.

